# Evaluation of Pediatric Patients Admitted to the Emergency Department Due to Drug Intoxication

**DOI:** 10.7759/cureus.13366

**Published:** 2021-02-16

**Authors:** Huseyin C Halhalli, Tolga Uslu

**Affiliations:** 1 Emergency Medicine, University of Health Sciences, Kocaeli Derince Training and Research Hospital, Kocaeli, TUR

**Keywords:** epidemiology, poisoning, child, drug, intoxication

## Abstract

Introduction: Pediatric intoxication cases are one of the important emergency room admissions. Early diagnosis and treatment are important in reducing morbidity and mortality. The prevalence and exposure types of pediatric intoxications have social and regional differences. In this study, we aimed to retrospectively analyze the demographic and epidemiological characteristics, clinical course, and prognosis of patients admitted to our Emergency Medicine Clinic due to poisoning.

Materials and Methods: In our study, the files of patients under 18 who were considered drug intoxicated and admitted to the ED were scanned retrospectively between 01.01.2018 and 31.12.2019. The cases were grouped as preschool (0-4 years old), school-age (4-12 years old), and adolescent (12-18 years old). The relationship between the frequency of emergency admissions of the patients, the variety of active ingredients, the amount taken, the need for hospitalization according to the active ingredients, admission times, arrival time to the emergency room, age, and gender were analyzed.

Results: When the distributions by age groups are examined, most of the patients constitute the two-year-old (22%). Besides, it was found that among the age groups, there were more girls than boys in the 12-18 age group. A statistically significant difference was found between the frequency of active substance intake and gender differences according to age groups (p <0.001, p <0.001, respectively). However, no statistically significant relationship was found between age groups and seasonal admission frequencies (p = 0.055).

Conclusions: Our study found a statistically significant difference in the amount of active substance taken or exposed to by age groups and gender. However, this finding does not explain the effect of active substance intake or exposure and gender on mortality and morbidity, probably due to the limited sample size.

## Introduction

In the pediatric age group, poisoning is a cause of serious morbidity and mortality. However, it has features that can be easily prevented with some simple measures. Among children and adolescents under 20 years of age, acute poisoning is associated with approximately 45,000 deaths per year, according to World Health Organization (WHO) data [1]. Also, acute poisoning cases concern a much larger universe due to various morbidities, except for severe consequences. Poisoning has an important place in childhood emergency room admissions, and more than 50% of the patients who reported to poison counseling centers are children under the age of five [2]. In the pediatric age group, intoxication mechanisms are mostly observed due to accidents and suicide, with an annual incidence of 0.02%-0.93% in developed countries [3]. Drug poisoning, which is one of the most common causes of emergency room admissions under 18, is also an important public health and socioeconomic problem. Early diagnosis and treatment play an important role in reducing morbidity and mortality [4].

Substances that cause acute toxicity in intoxications due to accident and suicide in the pediatric age group; vary according to the region of residence, socioeconomic level of the community, education level, and seasons. For this reason, it is important to determine the characteristics of the factors that cause general intoxication in each geographical location and region and to take measures accordingly. Considering the substances that cause poisoning in general, pharmacological agents (43.4%), nutrients and plants (21.8%), insecticides and pesticides (8.5%), carbon monoxide (8%), cleaning agents (7.2%), and hydrocarbons (5.4%). The cause of 5.7% of intoxications cannot be determined. Drug intoxications are the most common drugs with analgesics, antipyretics, and toxic effects on the nervous system [5].

It is important to anticipate broad pharmacological possible factors and their potential effects in the rapid diagnosis and treatment approach in cases presenting with drug poisoning. Especially in the pediatric age group, sometimes it may not be determined exactly what the agent is and the extent of exposure. For this reason, it is valuable to know the most common factors, especially in terms of investigating specific symptoms and guiding laboratory studies. In addition to the need for the tests and treatment of patients admitted to emergency services with drug poisoning as soon as possible, these patients should also be considered as forensic cases. Because of the existence of abuse and negligence in this age group and revealing it, if any, can be life-saving. However, the necessary information should be given to the family during discharge against the risk of recurrence of drug intoxications. Also, considering the frequency of drug poisonings, it is recommended to establish appropriate in-hospital organizations considering the cost to the country's economy [6]. To provide medical care at the most practical level, it is important to know the rates of applying to the ED, application methods, ages, and admission time intervals.

Studies aimed at determining the medical and demographic data of individuals who have been victims of poisoning are of great importance in reducing the mortality and morbidity experienced. There are many studies on this subject. In this study, we aimed to retrospectively analyze the demographic and epidemiological characteristics, clinical course, and prognosis of patients admitted to our Emergency Medicine Clinic due to poisoning.

## Materials and methods

Our study was conducted in an ED, a tertiary care center, to which 400,000 patients apply annually. Between 01.01.2018 and 31.12.2019, the files of patients under 18 who were considered drug intoxicated and admitted to the ED were scanned, each day to be grouped separately. In the screening, electronic data system records and patient files of the patients were examined, and the age, gender, time of day, how long after the drug intake, the active agent or agents causing poisoning, whether hospitalization to the service or ICU was required were determined and recorded. The cases were grouped as preschool (0-4 years old), school-age (4-12 years old), and adolescent (12-18 years old). With this data set, the frequency of ED admissions of the patients determined, the variety of active agents, the amount taken, whether there is a relationship between the active agents and the need for hospitalization. Between these times, there are more frequent admissions, the difference between the active agents' intake and admission time to the ED, and the patient's age and gender.

Statistical analysis

Statistical analysis was performed using the Windows SPSS 22 software package. The normality of the distribution of continuous and non-continuous variables was tested using the Kolmogorov-Smirnov test. Categorical variables Chi-square test; Parametric variables were compared using Student's t-test. Values are expressed as mean, standard deviation, and percentage.

Ethics committee approval

Local Ethics Committee approval was obtained for the research on 18.11.2019 with the protocol number 2019-124.

## Results

Out of 387 patients who applied with drug poisoning ICD code (X.44) between 01.01.2018 and 31.12.2019, 241 patients whose information required for our study were determined in the electronic data system, and other patient records were included in the study. While 65.6% of these were female, 34.4% were male. The mean age of the patients included in the study was found to be 4 ± 6.47 years. Among the cases, we found that the 0-3 age group was the group with the most frequent drug intoxication. The second most common was the 12-18 age group with 39.8%. There was no statistical difference in terms of seasonal admission frequency. Besides, it was found that 51% (123) of the patients presented between 17 and 24 hours (Table 1).

**Table 1:** Demographic characteristics of patients.

	n=241	%
Gender		
Female	158	65.6%
Male	83	34.4%
Age groups		
0-4	103	42.7%
4-12	42	17.4%
12-18	96	39.8%
Seasons		
Spring	60	24.9%
Summer	64	26.6%
Autumn	54	22.4%
Winter	63	26.1%
Admission time to the intake		
1-7	24	10.0%
8-16	94	39%
17-24	123	51%

We found that 170 (73%) of the cases took a single active agent. Sixty-three of the patients (27%) had more than one active agent intake. A statistically significant difference was found between the frequency of active agent intake and gender differences according to age groups (p <0.001 and p <0.001, respectively). However, no statistically significant relationship was found between age groups and seasonal application frequencies (p = 0.055) (Table 2). The active agent in eight of the cases could not be determined.

**Table 2:** Age groups according to the number of drug active agents, gender, and seasonal differences.

	Age groups (years)			Total	p
	0-4	4-12	12-18		
	(n=103)	(n=42)	(n=96)		
Drug active agents					
1	83 (48.8%)	40 (23.5%)	47 (27.6%)	170 (73%)	<0.001
2	7 (20%)	0 (0%)	28 (80%)	35 (15%)	
3	4 (28.6%)	2 (14.3%)	8 (57.1%)	14 (6%)	
4	3 (33.3%)	0 (0%)	6 (66.7%)	9 (3.9%)	
5 and over	0 (0%)	0 (0%)	5 (100%)	5 (2.1%)	
Total	97 (41.6%)	42 (18%)	94 (40.3%)	233 (100%)	
Gender					
Female	56 (54.4%)	21 (50%)	81 (84.4%)	158 (65.6%)	<0.001
Male	47 (45.6%)	21 (50%)	15 (15.6%)	83 (34.4%)	
Seasons					
Spring	23	19	22	64	0.055
Summer	29	7	18	54	
Autumn	28	8	27	63	
Winter	23	8	29	60	
Total	103	42	96	241	

**Figure 1:**
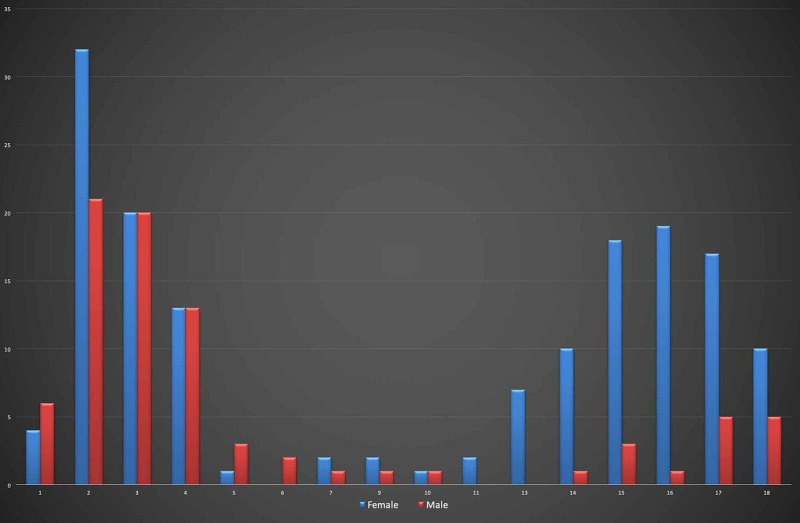
Frequency of application by age and gender.

When the distributions by age are examined, most of the patients are in the 0-3 age group, mostly two-year-old patients (22%). Besides, it was found that the 12-18 age group was higher in females than in males (Figure 1).

When the application times of the patients to the ED after taking the medication were examined, it was found that the patients presented to the ED within 15 minutes at the earliest, the average application time was 125.68 minutes, and 79.4% of the patients presented to the emergency service within the first two hours (Table 3).

**Table 3:** Application times to the ED after intoxication.

Emergency admission period after intoxication (n=133)	n (%)	Cumulative percent
First 1 hour	88 (62.4%)	62.4%
1-2 hours	24 (17%)	79.4%
2-3 hours	9 (3.7%)	85.8%
3-4 hours	5 (6.4%)	88.4%
4 and above	15 (16.5%)	100%

Some 98.8% of the patients were hospitalized, and 95.9% of the hospitalized patients were followed up in the service, and 2.9% in the ICU.

Some 66.3% of the patients were admitted to the ED with a single drug intake history.

**Figure 2:**
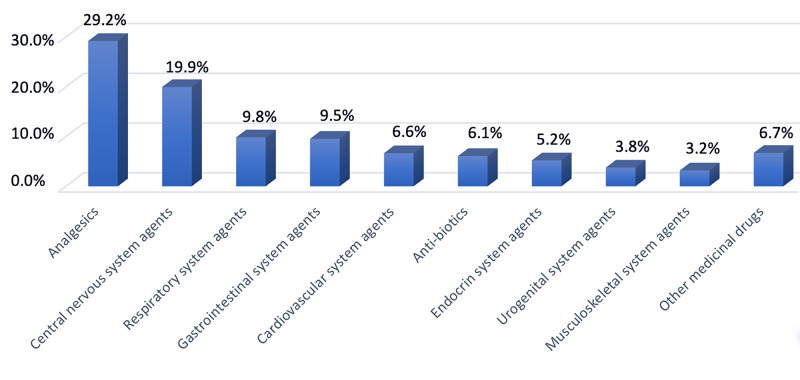
Distribution of medicinal poisoning.

Considering the percentage of intake according to drug types, the most common drugs are analgesics. Some 29.2% of all patients who took one or more drugs were poisoned with analgesic derivative drugs. Central nervous system drugs follow analgesics with 19.9% and respiratory system drugs with 9.8% (Figure 2).

**Figure 3:**
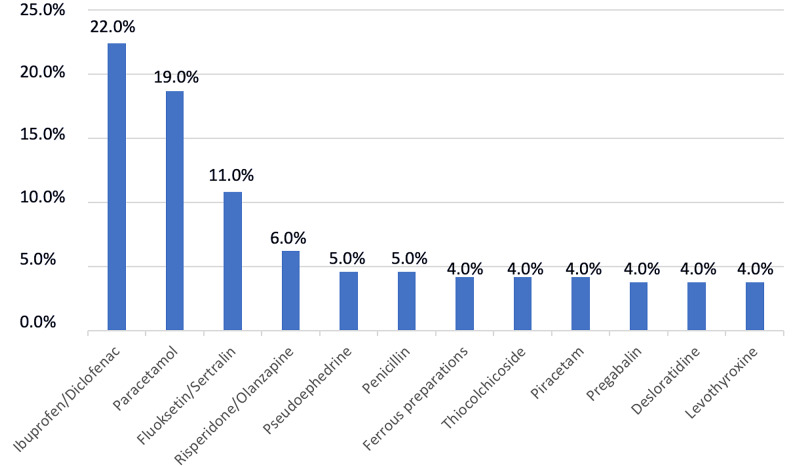
Most frequent agents causing poisonings.

When the most frequently taken drugs were examined according to the active agent, it was observed that 22% of all patients took ibuprofen/diclofenac, 19% paracetamol, and 11% fluoxetine/sertraline type drugs (Figure 3).

## Discussion

A total of 241 cases evaluated in our study constitute approximately 0.06% of all ED admissions. However, this rate seems to be compatible with the literature, considering that it covers pediatric cases and all ED cases where adult patients are cared for and only intoxications with pharmacological agents [7-8]. Although drug intoxications are one of the frequent reasons for ED, unconscious exposure specific to the pediatric age group and the inability to determine the extent of exposure may include differences. In many areas, there are general ED where children and adults are cared for together. For this reason, the data of the EDs evaluating all age groups, except for pediatric ED where only pediatric patients are evaluated, are valuable in terms of planning practices that will improve the quality of patient care. We aimed to determine the patients' demographic characteristics, the substances exposed and the ED application time intervals, and the ED admission times after exposure. In our study, where we evaluated the pediatric age group drug intoxications, 65.6% of the cases were female. The female/male ratio was found to be 1.90. When the literature is examined, it is seen that this ratio is generally compatible with the literature [8-9]. There are other studies in which the number of male patients (53.6%) was higher, as in the study by Gokalp [8, 10]. There are also studies in which female gender is more prevalent, as in Tüfekçi et al.'s study [11]. These differences may be related to the limitation of the study population. Studies dealing with national data may be more instructive in this regard [12]. Our study also seems compatible with the literature in this respect.

The mean value of the patients' ages included in our study was determined as 8.24 ± 6.47. Among the cases, we found that the 0-3 age group was the group with 97 (41.6%) cases with the most frequent drug intoxication. The second most common was the 12-18 age group with 94 (40.3%) cases. Similar to our findings, two peaks occur in the preschool and adolescent periods in studies using similar age grouping in the literature [12-13]. Although not evaluated separately in our study, accidental causes in the 0-3 age group and suicidal causes in the 12-18 age group should be considered more prominently. As in our study, the higher rate of female gender in the 12-18 age group seems consistent with the literature [7]. When we examined the seasonal relationship of age groups, we observed an increase in the 12-18 age group in the autumn and winter months. This difference is not statistically significant, but there are conflicting literature results [2, 13]. These differences can be explained by the fact that age groups were ignored in evaluating seasonal differences in other studies. For this reason, our study is original in that it shows that there is a statistically significant difference in terms of gender in pediatric intoxication cases above the age of 12 years. Although there is no statistically significant difference in season, it shows that the frequency of intoxication increases in the autumn and winter months.

Most of the cases of pediatric drug poisoning presenting to the ED in the evening (17:00-23:59). Studies on this subject in the literature generally support our findings [14-15].

Our study found that 170 (73%) of the cases took a single active substance. More than one active substance intake increases in older ages. This situation can be interpreted as more accidental intoxication cases in the 0-3 age group and an increasing number of suicidal intoxication cases in older ages [7]. As the age groups grow, both the number of factors taken and the agent's amount increase. Our study is original as a study showing the number of factors taken or exposed by age groups.

Eighty-eight (62.4%) of 141 patients, in whom the difference between admission to the ED after taking medication, was admitted within the first hour. In similar studies, this rate was between 18.8% and 45% [16-17]. The rate we have found is maybe because the center where the study was conducted was an urban area, and the transportation facilities are easy, and all patients' data cannot be determined. However, one of the most important factors affecting this situation is that intake or exposure is not always determined, especially at younger ages, especially in the pediatric age period.

We found that almost all of the patients (98.8%) included in the study were hospitalized, and 2.9% of them required intensive care. The level of need for intensive care seems to be compatible with the literature [12]. However, the high rate of hospitalization may be attributed to local reasons. One of the reasons for the high rate of hospitalization decision can be explained by the low reliability of the anamnesis in the pediatric age group. Therefore, the physician's decision may tend to be kept under observation for a longer period.

Some 66.3% of the patients were admitted to the ED with a single drug intake history.

Considering the percentage of intake according to drug types, the most common drugs are analgesics. This finding is consistent with the literature [12]. Some 29.2% of all patients who took one or more drugs were poisoned with analgesic derivative drugs. Central nervous system drugs follow this with 19.9% and respiratory system drugs with 9.8%. The reason why analgesic drugs are detected more can be explained by their more frequent use, being easily accessible, and less careful about restricting children's transportation by being seen as harmless by parents. This result, which is frequently mentioned in the literature, is important in emphasizing awareness-raising studies. Families should not keep all kinds of drugs, including analgesics, in places uncontrollably accessible to the pediatric age group.

Our study has several limitations. Primarily, our study is a retrospective, single-center study. The data used in the analyses were created by examining the electronic data system and patient files. For this reason, some of the drug intoxication cases may not be evaluated. Although vital signs, complaints, and physical examination findings of the patients are valuable in determining the severity of intoxication cases, they were not evaluated in our study. Studies involving larger numbers of patients to be conducted on this subject may affect our findings' importance.

## Conclusions

Our study found a statistically significant difference between the number of active agents taken or exposed to and the number of active agents according to age groups. This difference cannot explain the effects of age groups on both gender differences and mortality and morbidity. More comprehensive studies are needed on age grouping.
